# Intrinsic Nature of Stochastic Domain Wall Pinning Phenomena in Magnetic Nanowire Devices

**DOI:** 10.1038/srep13279

**Published:** 2015-08-25

**Authors:** T. J. Hayward

**Affiliations:** 1 Department of Materials Science and Engineering, University of Sheffield, UK

## Abstract

Finite temperature micromagnetic simulations are used to probe stochastic domain wall pinning behaviours in magnetic nanowire devices. By exploring field-induced propagation both below and above the Walker breakdown field it is shown that all experimentally observed phenomena can be comprehensively explained by the influence of thermal perturbations on the domain walls’ magnetisation dynamics. Nanowires with finite edge roughness are also investigated, and these demonstrate how this additional form of disorder couples with thermal perturbations to significantly enhance stochasticity. Cumulatively, these results indicate that stochastic pinning is an intrinsic feature of DW behaviour at finite temperatures, and would not be suppressed even in hypothetical systems where initial DW states and experimental parameters were perfectly defined.

Understanding and controlling stochastic effects in nanomagnetic systems is an area of major research interest due to the critical importance of these effects to the performance of data storage technologies.

For simple systems, where a single energy barrier separates two magnetisation states, the effects of thermal perturbations can be modelled using the Arrhenius-Néel law^1^,^2^,^3^,^4^. However, for more complex mesoscopic systems that exhibit larger numbers of stable states and multiple transitional paths, effective modelling becomes much more difficult. In this regime, magnetisation states and switching behaviour are best modelled using micromagnetic simulations. However, thermal effects can manifest on relatively long timescales, and so detailed predictions of stochastic effects using finite temperature micromagnetics are generally impossible.

Of particular interest in the mesoscopic regime is the behaviour of domain walls (DWs) in planar magnetic nanowires^5^. Logic^6^,^7^ and memory^8^ devices based on their pinning and propagation have great technological potential, but development has been inhibited, in part by problems of stochasticity. Experimental observations of these effects can be classified into three broad categories:

1. In nominally defect free nanowires, DW transmission is probabilistic even for fields much greater than those required to initiate DW propagation^9^,^10^.
2. DWs pinned at artificial defect sites have complex, multi-mode depinning field distributions^11^,^12^,^13^,^14^,^15^.
3. DWs pass probabilistically through defect sites even when under the influence of applied fields that would not be expected to be able to induce depinning^13^,^16^,^17^.

Experimental results have shown a strong link between the onset of these stochastic phenomena and the Walker breakdown (WB) field^18^, above which the structures of DWs oscillate during propagation^19^. However, it has not yet been comprehensively proven whether the ultimate origin of these stochastic behaviours relates to systems having poorly defined initial conditions (e.g. initial DW structure and position), or reflects an exquisite sensitivity of their magnetisation dynamics to small perturbations, such thermal fluctuations.

In this paper we use finite temperature micromagnetic simulations to show that the full range of stochastic phenomena that have been observed experimentally in DW devices can be explained by the dynamical interplay between Walker breakdown phenomena and thermal fluctuations. Our work indicates that stochasticity is an intrinsic feature of DW motion at finite temperatures, and would not be suppressed even in hypothetical systems where initial DW states and experimental parameters were perfectly defined. The dynamical origin of these effects also means that DW pinning is an unusual system where thermally-induced stochasticity can be directly modelled using numerical micromagnetic techniques, making such simulations an excellent test-bed for examining approaches to controlling stochastic behaviours.

## Results

### Field-dependent dynamics

**Figure 1:**
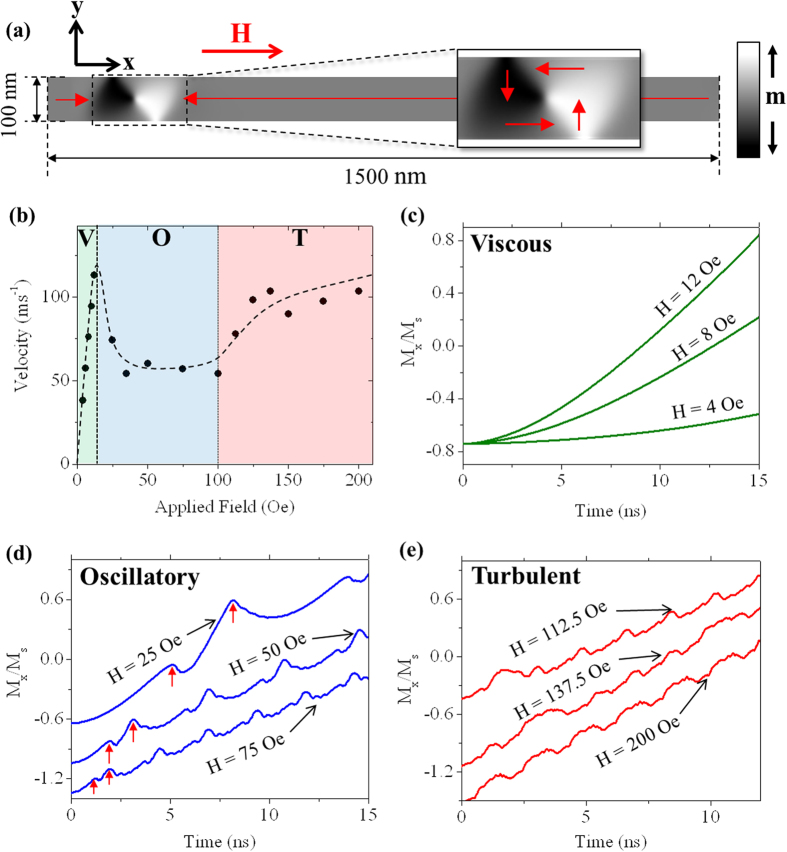
Field dependent propagation behaviour of DWs at T = 0 K. (**a**) Diagram showing the geometry of the simulated nanowire system. The initial DW configuration (ACW VDW) is also shown. (**b**) Variation of DW velocity as a function of applied field. The dashed line is a guide to the eyes. The three regimes of DW motion are indicated by the shaded regions: V = viscous, O = oscillatory, T = turbulent. (**c**) M_x_/M_s_ vs time plots for applied fields in the viscous regime of motion. (**d**) M_x_/M_s_ vs time plots for applied fields in the oscillatory regime of motion. The red arrows indicate the first VDW-TDW and TDW-VDW transitions in each trace. (**e**) M_x_/M_s_ vs time plots for applied fields in the turbulent regime of motion. The plots in (**d**) and (**e**) have been distributed vertically to improve legibility.

DW motion was simulated in a Ni_80_Fe_20_ nanowire with width w = 100 nm, thickness t = 20 nm and length l = 1500 nm. To understand the basic dynamics of the DWs, we initially simulated DW propagation at 0 K with applied fields in the range 2–200 Oe. An image illustrating the nanowire geometry and initial DW structure is shown in Figure 1(a).

Figure 1(b) shows how the velocity of the DWs varied over the applied field range. The data shows the characteristic shape expected for DWs in planar, soft ferromagnetic nanowires^20^, with three clear regimes of DW motion:

1. H < 15 Oe. Below the WB field (H_WB _= 15 Oe) the domain wall velocity increased linearly with applied field (**Viscous regime**).
2. 15 Oe < H < 100 Oe. Above the Walker breakdown field the domain velocity dropped due to the onset of oscillations in its internal magnetisation structure, and remained relatively constant over a large range of applied fields (**Oscillatory regime**).
3. H > 100 Oe. Beyond a further critical field the DW velocity again began to increase with applied field (**Turbulent regime**).

In the following we will analyse DW dynamics in each of these regimes in detail.

**Figure 2:**
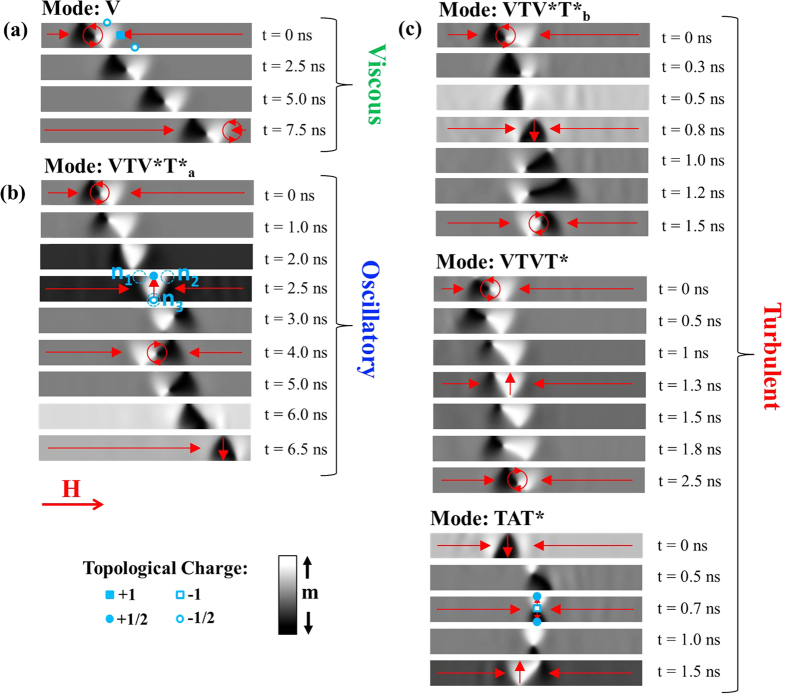
Results of micromagnetic simulations showing the modes of DW propagation observed in the (**a**) viscous, (**b**) oscillatory and (**c**) turbulent regimes of motion. The symbols placed on the figures indicate the distributions of topological charge in the vortex, transverse and anti-vortex DWs. Figure (**b**) also indicates the three possible locations (n_1_, n_2_, n_3_) in a transverse DW where the nucleation of a vortex/anti-vortex core can occur.

Figure 1(c) presents plots of M_x_/M_s_ against time for several fields within the viscous regime of DW motion. The DWs initially accelerated before propagating at a constant velocity. Figure 2(a) illustrates DW dynamics within this regime (Mode: V). The DWs propagated with relatively rigid magnetisation structures, as expected for propagation fields below H_WB_^18^.

Figure 1(d) presents plots of M_x_/M_s_ against time for fields within the oscillatory regime of DW motion above H_WB_. At all fields a periodic pattern was observed, with two characteristic peaks (marked by red arrows Figure), each of which corresponded to the onset of a short period of retrograde motion of the DW. The period of the oscillation decreased as the value of the applied field increased (although the DW velocity remained relatively constant).

Figure 2(b) illustrates DW dynamics within this regime (Mode: VTV*T*_a_). The basic dynamics of the WB mode consisted of an oscillation between vortex (VDW) and transverse (TDW) DW structure with the chirality of the DWs alternating between transitions (e.g. anticlockwise (ACW) VDW – TDW UP – clockwise (CW) VDW – TDW DOWN)^19^. The polarity of the VDWs’ cores also alternated, with clockwise VDWs having “up” core polarisation and anti-clockwise VDWs having “down” core polarisation. As the combination of core polarisation and chirality controls the trajectory of a vortex core^21^, this meant that the vortex cores always underwent a spell of retrograde motion, before resuming forward propagation and annihilating to form a new TDW. The TDWs also underwent a brief period of retrograde motion following their formation, completing the double peaked form exhibited by the M_x_/M_s_ plots.

In order to contrast this mode of WB with those that will be described later it is helpful to consider DWs in terms of elementary topological defects, as introduced by Tchernyshov *et. al*.^22^. In this model a TDW transverse DW consists of two topological defects with half integer winding numbers (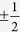) with the defect with positive winding number occurring at the wider side of the triangular-shaped DW and the negative defect being contained at the triangle’s apex (Figure 2(b)). A VDW consists of a +1 defect at the vortex core, and two negative half integer defects at the nanowire’s edges (Figure 2(a)). An anti-vortex DW consists of a -1 defect at the core and two positive half integer defects at the nanowire’s edges (Figure 2(c)).

As pointed out by Kim *et al*.^23^, there are three points in a TDW at which a vortex/antivortex core may nucleate. These are labelled n_1_, n_2_, n_3_ in Figure 2(b). Transformations of DW type must conserve total DW charge and thus a vortex core can only nucleate at points n_1_ and n_2_, while anti-vortices may only nucleate at point n_3_. In the case of vortex nucleation, the pre-existing TDW structure forms one half of the new VDW and thus the nucleation site, which lies either behind (n_1_) or in front (n_2_) of this, dictates the chirality of the new VDW. Nucleation of a vortex or antivortex core is preceded by the development of a strong, localised, out-of-plane magnetisation component. Mode VTV*T*_a_ can be characterised by vortex cores nucleating at points n_2_ of an initial TDW, and then annihilating at the trailing 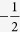 defect of the VDW to form a new TDW.

Figure 1(e) presents plots of M_x_/M_s_ against time within the turbulent regime of regime of motion. Here, the shapes of the plots were substantially more complex, and lacked the consistent, periodic profile observed in the oscillatory regime. Analysis of the simulated magnetisation dynamics indicated that this was because a total of three different WB modes could occur, with the dynamics at a given field representing either a single one, or a concatenation of several.

Details of these WB modes are illustrated in Figure 2(c), Mode VTV*T*_b_ was similar to that observed in the oscillatory regime, with the DW structure oscillating between VDW and TDW form and the chirality of the DWs also alternating in each cycle. However, vortex cores now nucleated at points n_1_ of the TDWs and annihilated at the leading 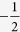 defects of the VDW, and thus the overall motion included substantially less retrograde motion than was observed in the oscillatory regime. Accordingly, the core polarisations of the vortex DWs were opposite to that in the previously described mode (clockwise = “down”, anticlockwise = “up”).

Mode VTVT* again consisted of an oscillation between vortex and transverse DW structure. However in this mode vortex chirality was conserved as in ref. 24, due to the nucleation of vortex cores occurring at the same points of the DWs as the previous cores had annihilated (i.e. *either* point n_1_ or n_2_). The overall effect was that a vortex core appeared to “bounce” from one edge of the nanowire to the other while retaining its chirality. The vortex core polarisation alternated as in the other observed modes.

Mode TAT* differed from the previously described modes in that it did not involve VDW structure. Here, transitions between TDWs of opposite chirality were mediated by the nucleation of anti-vortex cores from point n_3_. These propagated across the widths of the nanowires before annihilating at 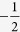 defects on the nanowires’ opposite edges. We note that this WB mode has previously been observed in thinner nanowire systems where TDW structure is favoured^19^.

It is notable that all of the WB modes observed involved periodic oscillations between transverse DWs of opposite chiralities. Therefore, the modes differed primarily in the way in which these transitions occurred, involving either VDWs with both CW and ACW chiralities (VTV*T*_a_, VTV*T*_b_), a single chirality (VTVT*) or anti-vortices (TAT*). In turn, these differences were related to the details of the magnetisation dynamics, with the WB mode exhibited being determined by whether the transitions out of TDW states occurred by nucleations at points n_1_, n_2_ or n_3_. This was in contrast to the well-defined nucleation behaviour observed in the oscillatory regime, where vortices always nucleated at site n_2_. Clearly, the DW dynamics in the turbulent regime were sufficiently complex and ill-defined that the site favoured for vortex/anti-vortex nucleation varied from cycle-to-cycle.

All of the relatively complex dynamics observed in the turbulent regime (Figure 1(e)) can be understood within the above framework. For example, the dynamics at 112.5 Oe consisted of modes VTV*T*_b_ and TAT*, while those at 137.5 Oe consisted of a combination of modes VTV*T*_b_ and VTVT*. The dynamics at 200 Oe occurred solely by mode VTVT*.

### Effects of thermal perturbations

Having established the basic field-dependent dynamics of the DWs we performed simulations at 300 K to probe how random thermal perturbations modified DW behaviour in each of the regimes.

**Figure 3:**
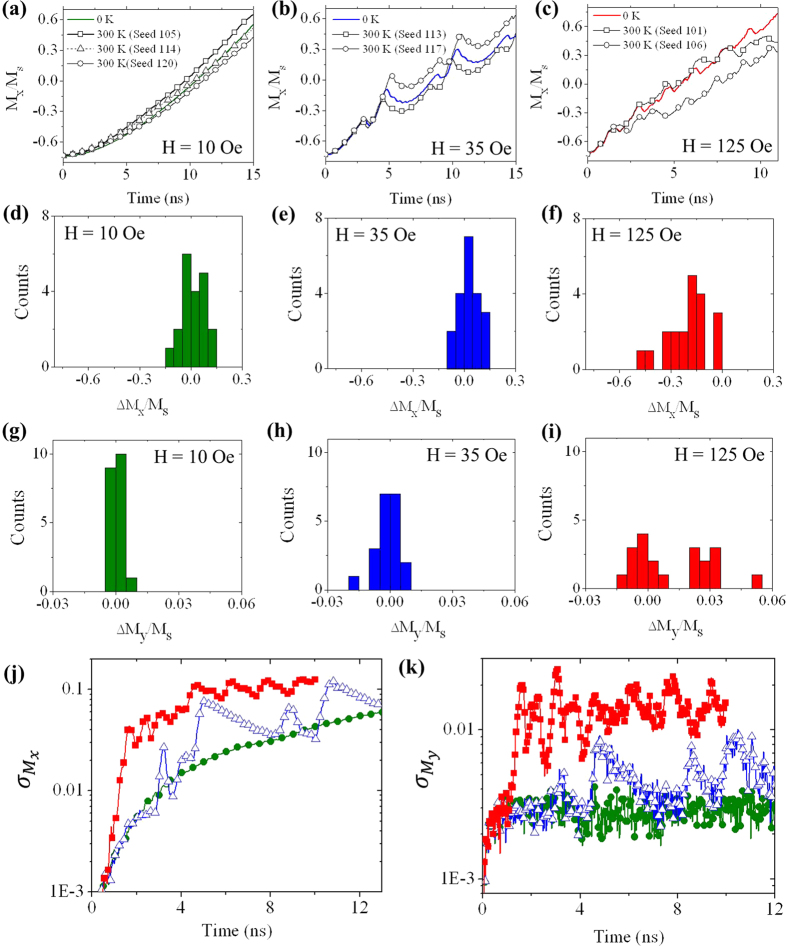
Propagation behaviour of DWs at T = 300 K. (**a**–**c**) M_x_/M_s_ vs time plots for the viscous, oscillatory and turbulent regimes. In each plot, data is shown for T = 0 K and for several seed values at T = 300 K. (**d**–**f**) Histograms of ΔM_x_/M_s_ after 1 μm of DW propagation for the viscous, oscilliatory and turbulent regimes. (**g**–**i)** Histograms of ΔM_y_/M_s_ after 1 μm of DW propagation for the viscous, oscillatory and turbulent regimes. (**j**) Variation of 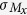 with time for all three regimes of DW motion. (Viscous = closed circles, Oscillatory = open triangles, Turbulent = closed squares). (k) Variation of 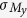
with time for all three regimes of DW motion.

Figure 3(a) presents M_x_/M_s_ against time plots for simulations performed in the viscous regime of motion (H = 10 Oe). Data are shown for three of the simulations performed at T = 300 K with different random seed values, as well for a simulation performed at 0 K. The addition of thermal perturbations caused, modest, moment-to-moment variations in the velocity of the DWs, such that after 15 ns of propagation DWs in simulations with differing seed values had propagated slightly different distances. Clearly, the relatively rigid structure of DWs in the viscous regime meant that thermal perturbations could only modestly influence their dynamics.

Figure 3(b) presents M_x_/M_s_ against time plots for simulations performed in the oscillatory regime of motion (H = 35 Oe). The effects of finite temperature were more marked here, with the T = 300 K simulations appearing to “decohere” from the T = 0 K data. Closer analysis of the plots revealed that this resulted from thermal perturbations interfering with the transitions between VDW and TDW structure, such that they were either delayed or expedited. This can be observed by closely examining the first full cycle of oscillation in Figure 3(b). Here, the first peak in the plot represents the transition from VDW to TDW structure, while the second represents the reverse transition. Both of these transitions occurred later in the T = 300 K, Seed 113 simulation than in the T = 0 K data. Conversely, both transitions occurred earlier in the T = 300 K, Seed 117 simulation. Clearly, the inherent instability of the DWs above WB allowed thermal perturbations to exert a strong influence on dynamics by interfering at delicately balanced points in the magnetisation dynamics (i.e. the nucleation and annihilation of a vortex cores), with the overall effect that the time taken for nucleations to complete successfully varied from one instance to the next.

Figure 3(c) presents M_x_/M_s_ against time plots for simulations performed in the turbulent regime of motion (H = 125 Oe). Here, dramatic differences in the shapes of the plots were observed. This was in contrast to the oscillatory regime, where the basic magnetisation dynamics were always maintained. Closer examination of the dynamics revealed that these dramatic changes in behaviour were caused by modifications to the sequence of WB modes the DWs exhibited: At T = 0 K only mode VTV*T*_b_ occurred, while in the finite temperature simulations all three accessible WB modes were observed during propagation. As noted earlier, the WB mode for a given oscillation was determined at the point of transition from TDW structure, with the location of vortex/anti-vortex core nucleation being the controlling factor. We deduce that in the turbulent regime thermal effects are not only able to interfere with the timing of the nucleation, as in the oscillatory regime, but also to randomise the location (n_1_, n_2_, n_3_) of vortex/anti-vortex nucleation, thus adding a random element to the WB modes exhibited.

To quantitatively study the effects of thermal perturbations on DW propagation we measured the deviation of the T = 300 K simulations from the T = 0 K simulations once DWs had propagated approximately 1 μm along the nanowire. For the viscous regime (H = 10 Oe), oscillatory (H = 35 Oe) and turbulent regimes (H = 125 Oe) this corresponded to times t = 13.4 ns, t = 13.6 nm and t = 7.8 ns respectively. For each finite temperature simulation we measured the deviation of both M_x_/M_s_ and M_y_/M_s_ from those in the equivalent 0 K simulation. The resulting values ΔM_x_/M_s_ and ΔM_y_/M_s_ represented the deviation of DW position and internal magnetisation structure.

Distributions of ΔM_x_/M_s_ and ΔM_y_/M_s_ for each of the propagation regimes are shown in Figure 3(d–i), while the standard deviation of M_x_/M_s_ and M_y_/M_s_ (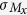
and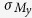) at each instant are shown in Table 1. The magnitude of these parameters can be understood by considering that a value of ΔM_x_/M_s _= ±0.13 represented a deviation of ±100 nm in DW position, while ΔM_y_/M_s _= ±0.038 was equivalent to the difference between VDW and TDW structure. The data show that after 1 μm of propagation, variability in both the position and structure of the DWs increased through the viscous → oscillatory → turbulent regimes. For fields in the turbulent regime, DW positions varied by over 300 nm, with the variability in ΔM_y_/M_s_ being consistent with DWs being instantaneously in completely different configurations.

We note that, due to the periodic transformations of DW structure that occurred in the oscillatory and turbulent regimes, examining variability at a fixed point has only limited validity. This is illustrated in Figure 3(j,k) which present values of 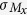 and 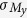
as a function of time for all three regimes. The data for both the oscillatory and turbulent regimes exhibit clear peaks, corresponding to transitions between DW types. At these points variability in the position and structure of the DWs was significantly enhanced (comparing for example the data at t = 13.6 ns and 10.5 ns for H = 35 Oe in Table 1). As these transitions do not occur in the viscous regime, the variability observed is much lower, and shows no periodic character.

### Stochastic Pinning Behaviour

**Figure 4:**
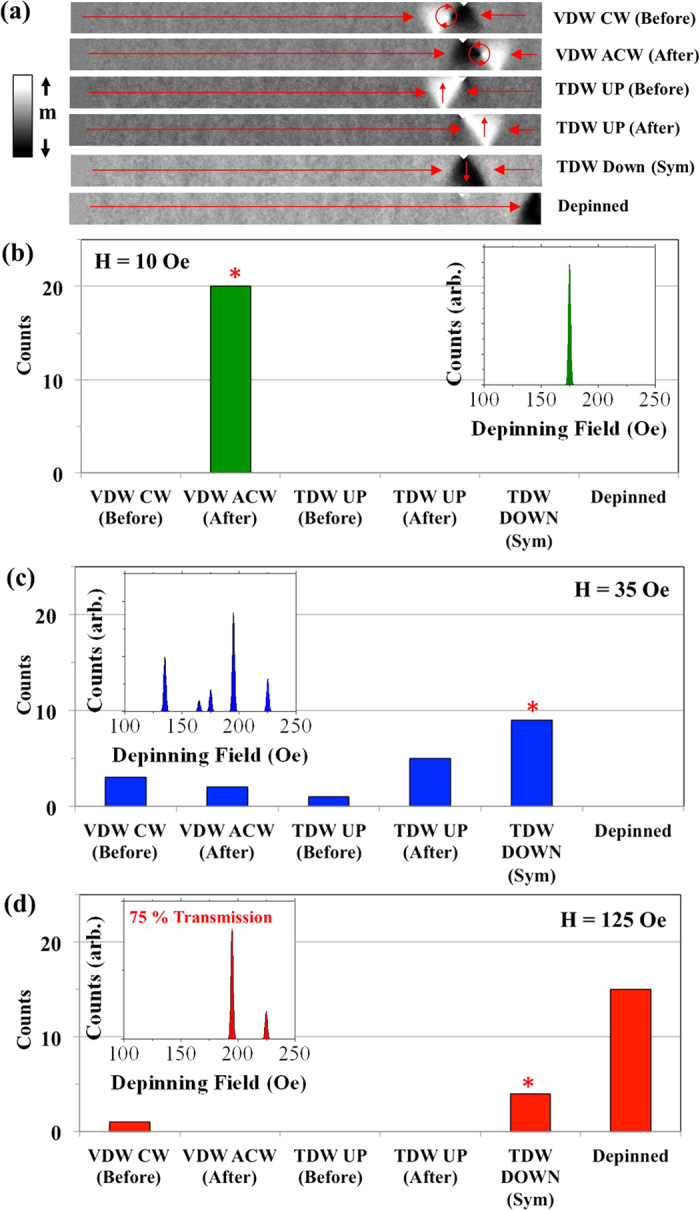
Stochastic pinning of DWs at an artificial defect site at T = 300 K. (**a**) Results of Micromagnetic simulations showing the configurations of DWs pinned at the artificial defect site. (**b**–**d**) Plots illustrating the distribution of pinned DW states for H = 10 Oe (Viscous regime), H = 35 Oe (Oscillatory regime) and H = 125 Oe (Turbulent regime). The star symbols indicate the pinned states observed in T = 0 K simulations. Derived depinning field distributions are shown inset in each figure.

Having analysed the effect of thermal perturbations on the free propagation of DWs we investigated their pinning behaviour by introducing a 25 nm wide, 12.5 nm deep triangular, notch-shaped defect site into the upper edge of the nanowire at a distance of 1.25 μm from its left-hand end. The geometry of the notch allows five distinct DW configurations to be pinned strongly, as shown in Figure 4(a). Quasi-static depinning fields were determined for each of these configurations and are listed in Table 2.

Figure 4(b–d) present the distributions of pinned DW states observed at T = 300 K in each of the three propagation regimes. These distributions were used with the depinning field values in Table 2 to derive the depinning field histograms shown on the right-hand side of the figures by assuming that each well-defined DW configuration would exhibit a Gaussian distribution of depinning fields with σ ~ 1 Oe.

Figure 4(b) presents the distribution of states observed for DWs propagating to the defect site in the viscous regime (H = 10 Oe). In all of the simulations the pinned DWs retained their initial ACW VDW structure, and thus the derived depinning field distribution consisted of a single peak. We note that this low level of stochasticity for DWs propagating below WB has also been observed experimentally^9^,^10^,^13^.

A much greater degree of stochasticity was observed in the oscillatory regime (Figure 4(c)). All five possible pinned DW states were observed, leading to a depinning distribution with five distinct peaks spanning a range of 90 Oe. Analysis of the magnetisation dynamics showed that this variety of states was formed because the “dephasing” of the transitions between DW states at finite temperature meant that each DW reached the notch at a slightly different point in its WB sequence, thus causing them to interact differently with the notches pinning potential and form different pinned states.

Increasing the field into the turbulent regime (Figure 4(d)) produced further changes in the distributions of pinned states. In 75% of the simulations the DWs were found to pass through the notch without pinning, despite the fact that the applied field (H = 125 Oe) was 10 Oe lower than the lowest depinning field value shown in Table 2 (TDW UP (after)). Several experimental studies have also observed that the fields required to cause DWs to pass dynamically through defect sites are lower than those required to depin DWs that have already become pinned^13^,^16^,^17^, and we suspect this is an example of the same phenomenon. In fact, performing additional T = 0 K simulations with fields in the range H = 110–140 Oe showed that pinning was extremely sensitive in this regime, with DWs passing the defect site at 60% of the fields simulated, with no obvious pattern to the distribution of fields at which pinning occurred. Clearly the ability of the notch to pin the DWs was highly sensitive to their configurations. In the T = 300 K simulations these varied due to the randomisation of the WB modes, hence the probabilistic nature of DW pinning. In the remaining 25% of simulations DWs were observed to pin in either a TDW DOWN (sym) or CW VDW (before) configuration, producing a depinning field histogram with two peaks. Notably these were the DW configurations with the highest depinning fields.

### Effect of Roughness

To investigate how additional disorder in the form of randomised defects affected finite temperature DW dynamics we performed simulations of propagation in nanowires with rough edge profiles.

**Figure 5:**
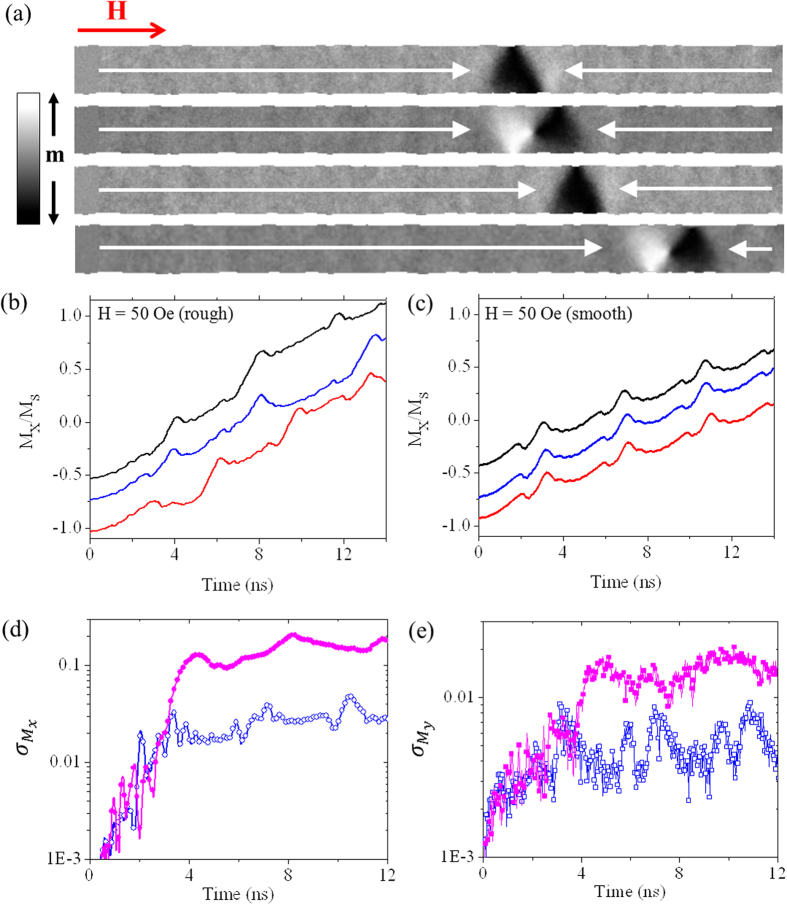
Comparison of the propagation and pinning behaviour of DWs in rough and smooth nanowires at T = 300 K. (**a**) Results of micromagnetic simulations showing the configurations of DWs that pinned during propagation through a rough nanowire at H = 50 Oe. (**b**) M_x_/M_s_ vs time plots illustrating the propagation of DWs through a rough nanowire at H = 50 Oe, T = 300 K. (**c**) M_x_/M_s_ vs time plots illustrating the propagation of DWs through a smooth nanowire at H = 50 Oe, T = 300 K. (**d**) Variation of 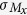 with time for propagation at H = 50 Oe in both rough (closed symbols) and smooth (open symbols) nanowires. (**d**) Variation of 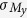 with time for propagation at H = 50 Oe in both rough (closed symbols) and smooth (open symbols) nanowires. The plots in (**b**,**c**) have been distributed vertically to improve legibility.

We performed the simulations with roughness amplitude factor λ = 20 nm and correlation length, σ = 1 nm. The edge profile generated by these parameters can be seen in Figure 5(a). We note that, in contrast to previous studies using finite element techniques^25^, the cubic finite difference mesh used in our simulations meant that the nanowire’s edge profile was rendered imperfectly. However, a large number of random defects sites were still created, making the approach suitable for our purposes.

T = 0 K simulations showed that no DW propagation occurred for H < 35 Oe in the rough nanowires, with the DWs remaining pinned at their initial location. For 35 Oe ≤ H < 70 Oe propagation appeared to be highly sensitive, with DWs pinning during propagation at some fields and propagating to the end of the nanowire at others. For H ≥ 70 Oe the DWs always propagated along the full length of the nanowire. We chose to examine finite temperature dynamics at H = 50 Oe, a field at which DWs propagated the full length of the nanowire at T = 0 K, but within the range of fields where pinning was possible. As a comparison we also performed both 0 K and T = 300 K simulations at the same field in a smooth nanowire. Here, the DWs exhibited WB mode VTV*T*_a_ as would be expected for a field within the oscillatory regime of DW motion.

In simulations performed at T = 300 K we found that propagation through the nanowire was probabilistic, with the DWs becoming pinned in 30% of the simulations performed. We observed a total of four different pinned DW configurations at a range of positions in the nanowire (Figure 5(a)). A common factor in the formation of these states was that they all pinned either at the point of, or shortly after, transitions between VDW and TDW structure. This suggests that the DWs pinned during the short periods of retrograde motion that occurred following transitions between DW types. We suspect that the loss of forward momentum at these points aided the pinning of the DWs^25^.

In addition to causing probabilistic propagation we also observed that the nanowire’s roughness had a significant effect on the DWs propagation dynamics. This is illustrated in Figure 5(b,c), which show M_x_ against time plots for simulations at T = 300 K in both the rough and smooth nanowires. In the case of the smooth nanowires (Figure 5(c)) the data showed the characteristic periodic shape associated with the VTV*T*_a_ WB mode (although close analysis showed that thermal perturbations could delay or expedite the nucleation/annihilation of vortex cores as in the simulations at H = 35 Oe). In contrast to this, the dynamics in the rough nanowires were more complex, and lacked a periodic profile. Analysis of the DW dynamics revealed the rough wires did not show a consistent WB mode. Rather, they underwent periods of dynamics characteristic of modes VTV*T*_a_, VTV*T*_b_ and VTVT* (i.e. vortex nucleation occurred at both points n_1_ and n_2_). We also note that the points at which transitions between DW states occurred were poorly defined in the rough wires, with TDW configurations slipping significant distances along the nanowires before the nucleation of vortex cores in some simulations, and DWs appearing to get briefly caught at defect sites in others. The poorly defined DW dynamics in the rough nanowires greatly enhanced the variability in DW position and structure, as can be seen in Figure 5(c,d) which contrast plots of 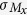 and 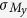 for the rough and smooth nanowires.

## Discussion

The results we have presented above show that thermal perturbations have a profound effect on DW dynamics, introducing significant uncertainty in the position and structure of DWs during their propagation, and in their pinning behaviour on encountering defect sites.

The underlying cause of these effects is the ability of thermal excitations to interfere at critical points in a DW’s magnetisation dynamics. In the low field, viscous propagation regime, where DWs propagate relatively rigidly, no such critical transitions occur and so the effects of thermal perturbations are minor. However, the nucleation and annihilation of vortex and anti-vortex cores that occur above WB are highly sensitive to thermal perturbations, and thus thermal effects are strong in both the oscillatory and turbulent regimes. Our simulations suggest that these effects are strongest in the turbulent regime, where the inherent instability of the DWs allows small perturbations to produce dramatic differences between the magnetisation dynamics of DWs propagating under the same external conditions. However, even in the oscillatory regime of motion, where the overall form of the magnetisation dynamics is basically well-defined, the ability of thermal perturbations to delay/expedite transitions between DW states can make pinning highly stochastic.

In systems with significant edge roughness, we have observed a large increase in the thermally-induced uncertainty in the position and structure of propagating DWs. This has some important implications. Firstly, it suggests that the interaction of DWs with defect sites will not only be a product of the geometry of that defect, but of a nanowire as a whole. Secondly, the degree of stochasticity exhibited by DW systems will depend on whether DWs pass ballistically though the energy landscape presented by edge roughness, and thus on both the strength of the applied field, and the lithographic quality of the nanowires. Finally, our simulations show that edge roughness can cause variation in the WB modes exhibited by DWs, even in the oscillatory regime where the magnetisation dynamics are expected to be well-defined. Hence, it is important to consider lithographic quality when considering whether a system will exhibit dynamics that conserve DW chirality as demonstrated in ref. 24.

We note that our simulations reproduce all of the major features of stochastic DW behaviour observed experimentally: Our results show stochastic pinning behaviour to be supressed at very low propagation fields, below WB. This has been observed in a number of experimental studies where DW injection by current pulses through orthogonal current lines have allowed DWs to propagate at sufficiently low fields^9^,^10^,^13^. At higher propagation fields, similar to those typically required to inject DWs from a nucleation pad, we see DWs pin at artificial defect sites in a variety of magnetisation states, and thus exhibit multi-mode depinning field distributions. This is often considered to be the generalised behaviour of DW pinning at such defects, and has been observed in a large number of experimental studies^11^,^12^,^13^,^14^,^15^. We have also observed DWs probabilistically passing through defect sites at fields lower than those required to depin a static DW, a phenomena that has been observed in a variety of papers investigating dynamic pinning effects^13^,^16^,^17^. Finally we have observed probabilistic transmission of DWs through nanowires at fields greater than those required to initiate DW motion. Again, these effects have been observed in a number of high-quality experimental studies^9^,^10^.

Our results suggest that all of these stochastic behaviours are an intrinsic feature of DW dynamics at finite temperature, and would still be observed even in hypothetical systems where both initial DW states and external conditions could be perfectly defined. We note that does not imply that the effects observed in experimental studies are entirely of thermal origin; indeed, in the majority of studies initial DW structures and/or propagation fields will not be perfectly defined and may make significant contributions to stochasticity. However, even if one could optimise devices/experiments so that these parameters were highly regulated, our simulations suggest stochastic DW pinning would still occur.

A final point of interest is that, because the stochastic pinning effects in DW systems are of dynamic origin, they represent a rare case where the stochasticity observed in quasi-static experimental measurements can be directly modelled using micromagnetic simulations. This is of note because behaviours caused by conventional thermal activation typically take place on timescales much too long to be computationally accessible. Indeed, there is some evidence that thermally-activated transitions between DW states may also occur during DW depinning from notches^26^. Furthermore, other modelling^27^ and experimental studies^28^ have shown that DW pinning at small, roughness-related defect sites, can in-fact be a transient phenomena, with DWs thermally activating over energy barriers to continue their propagation. While these effects were not observed in this study, in real systems such phenomena will add additional layers of complexity on top of the dynamic effects we have modelled here.

It is important to consider some of the limitations of the modelling approach used. Performing finite temperature micromagnetic simulations on a discretised mesh has the effect off applying a “cut-off” to the spin wave spectrum at wavelengths equivalent to the cell dimensions. This can result in substantial errors. For example, calculations using mesh dimensions similar to those used here typically over-estimate Curie temperatures by around an order of magnitude unless material parameters are renormalized^29^.

At lower temperatures, close to room temperature, mesh size dependent effects are less substantial, and typically estimate quantitative equilibrium magnetisations to within a few % of the results of atomistic or renormalized calculations^29^,^30^,^31^. Furthermore, the results presented here model the effect of small perturbations on complex dynamics, rather than equilibrium configurations, and thus the quantitative validity of the calculations will depend strongly on how susceptible these magnetisation processes are to the influence of short wavelength excitations. To investigate this further it would be necessary to either simulate DW systems that are computationally accessible by both micromagnetic and atomistic techniques, or perform simulations using the stochastic Landau-Lifshitz-Bloch equation, which is valid at all temperatures^32^. In any case, finite discretisation typically causes the effects of thermal perturbations to be *underestimated*, and hence it is unlikely that the effects observed would be diminished when using more rigorous treatments.

## Conclusions

In this paper room-temperature micromagnetic simulations have been used to directly model stochastic DW pinning effects in magnetic nanowires. The results replicate all of the major stochastic behaviours that have been observed in experimental studies. This indicates that these effects are an intrinsic feature of DW behaviour at finite temperatures, originating in a delicate interplay between thermal perturbations and the complex magnetisation dynamics that DWs exhibit when propagating under fields in excess of the Walker breakdown field. A major implication of this is that stochastic effects in DW devices would not be suppressed even if initial DW states and operating parameters could be perfectly defined. To our knowledge this paper is the first work to directly address the question of whether the influence of thermal perturbations alone are strong enough to introduce substantial stochasticity in DW behaviour.

The results presented here are primarily phenomenological and further studies, in which larger numbers of statistics are examined, will be required to gain a more quantitative understanding of how temperature affects the variability of DW propagation in nanowire systems. However, the simulations can be considered to represent a “working model” of stochastic effects in nanowire devices, and will be a useful platform with which to test potential methods of controlling these effects.

There is also evidence in the data that DWs undergoing Walker breakdown at finite temperature may exhibit cyclostationary behaviour^33^, i.e. show periodic variations in their statistical characteristics (see for example the periodic peaks in 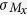 and 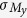 in Figure. 3(j,k)). Stochastic systems with cyclostationary properties have been previously been observed at a wide range of length and time-scales, ranging from the radio frequency emissions of distant stars and planets, to the climate of our own planet, to the signals observed in telecommunication systems. While further statistical analysis will be required to show conclusively that finite temperature DW dynamics have cyclostationary properties, it is entirely possible that they represent an example of these phenomena at nanosecond timescales and nanometer length scales.

## Methods

Micromagnetic simulations at both 0 K and 300 K were performed using the MUMAX^3^ package^34^ developed at Ghent University. Simulations were performed with a Heun solver, and in the finite temperature cases followed the method of Brown^35^ to introduce a random thermal effective field with properties determined by the fluctuation-dissipation theorem. The finite temperature simulations were performed with a fixed time step of 1 fs, while the 0 K simulations used an adaptive time step. Test simulations performed with a fixed time step of 0.5 fs showed identical behaviour to those performed with the higher time step. For each set of external parameters investigated at finite temperature, 20 individual simulations were performed with thermal seed values in the arbitrarily chosen range 101–120.

DW motion was simulated in a Ni_80_Fe_20_ nanowire with width w = 100 nm, thickness t = 20 nm and length l = 1500 nm. We used standard parameters for the material constants of NiFe: saturation magnetisation, M_s _= 860 kA/m and exchange stiffness, A = 13 pJ/m. The effects of the magnetic poles at the nanowires ends were subtracted using a standard function of the simulation package, making the DW dynamics equivalent to those in an infinite nanowire.

The profiles of nanowires with edge roughness were defined in terms of an amplitude factor, σ and a correlation length λ^25^. Node points were distributed along each edge of the nanowire spaced by λ. At each node a random displacement from the nominal edge position was generated using a normal distribution with standard deviation = σ. Finally, the full edge profile was interpolated using a cubic spline through the node points and inputted into the micromagnetic solver as an image mask.

Initial studies indicated that vortex DWs were favoured in the chosen nanowire geometry, having an energy ~8% lower than transverse DWs. Thus, to study DW motion we initialised a head-to-head (H2H) anticlockwise vortex DWs 200 nm from the nanowire’s left end. The vortex cores’ magnetisations were oriented along +z (up). Magnetic fields were then applied along the nanowires length to induce DW motion.

## Additional Information

**How to cite this article**: Hayward, T.J. Intrinsic Nature of Stochastic Domain Wall Pinning Phenomena in Magnetic Nanowire Devices. *Sci. Rep*. **5**, 13279; doi: 10.1038/srep13279 (2015).

## Figures and Tables

**Table 1:** Instantaneous values of 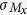 and 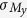 measured at propagation field H and time t.

	H (Oe)			
	10 Oe (t = 13.4 ns)	35 Oe (t = 13.6 ns)	35 Oe (t = 10.5 ns)	35 Oe (t = 7.8 ns)
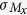	6.2 × 10^−2^	6.3 × 10^−2^	9.4 × 10^−2^	12.2 × 10^−2^
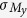	2.5 × 10^−3^	5.1 × 10^−3^	10 × 10^−3^	21 × 10^−3^

**Table 2:** Quasi-static depinning fields determined for the five pinned DW configurations shown in Figure 4(a).

DW Structure	CW VDW (Before)	ACW VDW (After)	TDW UP (Before)	TDW UP (After)	TDW DOWN (Sym)
Depinning Field (Oe)	225 ± 5	175 ± 5	165 ± 5	135 ± 1	195 ± 5
